# The sensitivity of different methods for detecting abnormalities in auditory nerve function

**DOI:** 10.1186/s12938-020-0750-2

**Published:** 2020-02-03

**Authors:** Tianhao Lu, Qiang Li, Chen Zhang, Min Chen, Zhengming Wang, Shufeng Li

**Affiliations:** 1 Department of Otolaryngology, Eye & ENT Hospital of Fudan University, 83 Fenyang Road, Shanghai, 200031 China; 2 NHC Key Laboratory of Hearing Medicine, Shanghai, China

**Keywords:** Electrically evoked compound action potential, Electrically evoked auditory brainstem response, Electrical stimulation, Different functional states of auditory nerve

## Abstract

**Background:**

Cochlear implants (CIs) have become important for the treatment of severe-to-profound sensorineural hearing loss (SNHL). Meanwhile, electrically evoked compound action potentials (ECAPs) and electrically evoked auditory brainstem responses (EABRs), which can be examined and evaluated with minimal patient cooperation, have become more reliable for tone measurement and speech recognition postoperatively. However, few studies have compared the electrophysiological characteristics of the auditory nerve using ECAPs and EABRs under different functional states of the auditory nerve (FSANs). We used guinea pig models in which six electrodes were implanted unilaterally with continuous electrical stimulation (ES) for 4 h. The amplitude growth functions (AGFs) of the alternating polarity ECAP (AP-ECAP) and forward-masking subtraction ECAP (FM-ECAP), as well as the EABR waves under “normal” and “abnormal” FSANs, were obtained.

**Results:**

Both the AP-ECAP and FM-ECAP thresholds were significantly higher than those measured by EABR under both “normal” FSAN and “abnormal” FSANs (*p* < 0.05). There was a significant difference in the slope values between electrodes 1 and 2 and electrodes 3 and 4 in terms of the AP-ECAP under the “abnormal” FSAN (*p* < 0.05). The threshold gaps between the AP-ECAP and FM-ECAP were significantly larger under the “abnormal” FSAN than under the “normal” FSAN (*p* < 0.05).

**Conclusions:**

Both of the ECAP thresholds were higher than the EABR thresholds. The AP-ECAP was more sensitive than the FM-ECAP under the “abnormal” FSAN.

## Background

Cochlear implants (CIs) are the most effective aids that can help people with sensorineural hearing loss (SNHL) achieve auditory reconstruction [1–3]. Postoperative examinations and evaluations are also important components of the treatment process. To clinically evaluate the level of hearing recovery after CI, subjective audiometry is often performed by collecting a large amount of data in a short time and then assessing the hearing status of the individuals with the implants daily [4, 5]. However, individuals need CIs at increasingly younger ages; these patients cannot accurately respond to subjective evaluation problems, and it is difficult for doctors to determine their comfort level during the CI adjustment process postoperatively [6]. Therefore, objective evaluations, which can be performed with minimal patient cooperation, are more reliable than subjective evaluations for tone measurement and speech recognition [7].

Electrically evoked compound action potentials (ECAPs) and electrically evoked auditory brainstem responses (EABRs) are both valuable in evaluating the functional states of the auditory nerve (FSAN), as they correspond to the responses of the auditory pathway evoked by intracochlear electrical stimulation [8–10]. ECAPs correspond to compound action potentials of the auditory nerve that are recorded through the electrodes of the cochlear implant [11], while EABRs correspond to the responses of the integrity of auditory pathways that are recorded via electrodes placed on the scalp [12]. Measuring ECAPs is currently one of the most commonly used objective intraoperative evaluation methods. At the end of an operation, the compound action potentials that are produced by auditory nerves and detected by the electrodes of the CIs are recorded to determine whether the individual has a spectrum disorder of auditory neuropathy, and the action potentials can act as a stimulation reference for the initial cochlear activation [13]. There are two common stimulus artefact reduction paradigms that are used to record ECAPs, i.e., alternating polarity ECAPs (AP-ECAPs) and forward-masking subtraction ECAPs (FM-ECAPs) [14, 15]. EABRs can be used to monitor intraoperative auditory function, which can allow us to determine whether a CI has been successfully implanted. Specifically, EABRs are stimulated by the processor and electrodes of the CI to mimic sound processing in the brainstem under ES, and the CI can be determined to be effective or not according to the waveform differentiation results [16].

Few studies have investigated the differences in electrophysiological characteristics between ECAPs and EABRs, and no studies have demonstrated a consistent relationship between these two measures [17, 18]. Several studies have also compared the electrophysiological characteristics of AP-ECAPs and FM-CPAPs [19–22]. AP-ECAPs were found to have smaller amplitudes, higher thresholds and steeper slopes than FM-ECAPs for cochlear devices but not for advanced bionic devices [19, 22]. However, these previous studies were conducted in human cochlear recipients with hearing loss, who exhibit a variety of different pathophysiological mechanisms. Therefore, their FSANs might be different, which may have an impact on the consistency of the results.

Previous research from our laboratory has shown that the continuous stimulation of charge-balanced biphasic pulses to the cochlea can significantly elevate the ECAP threshold in guinea pigs [23]. These findings indicate that acute electrical stimulation (ES) has an inhibitive effect on the excitability of the auditory nerve. We refer to this kind of inhibited excitability as an “abnormal” FSAN relative to the original level of excitability, namely, the “normal” FSAN.

To evaluate the sensitivity of different methods in detecting abnormalities in auditory nerve function, we established guinea pig models with “normal” and “abnormal” FSANs by simply implanting intracochlear electrodes with a specific density and administering continuous ES with a specific duration. Then, we compared the electrophysiological characteristics of the AP-ECAPs, FM-ECAPs and EABRs of the two FSANs.

## Results

**Fig. 1:**
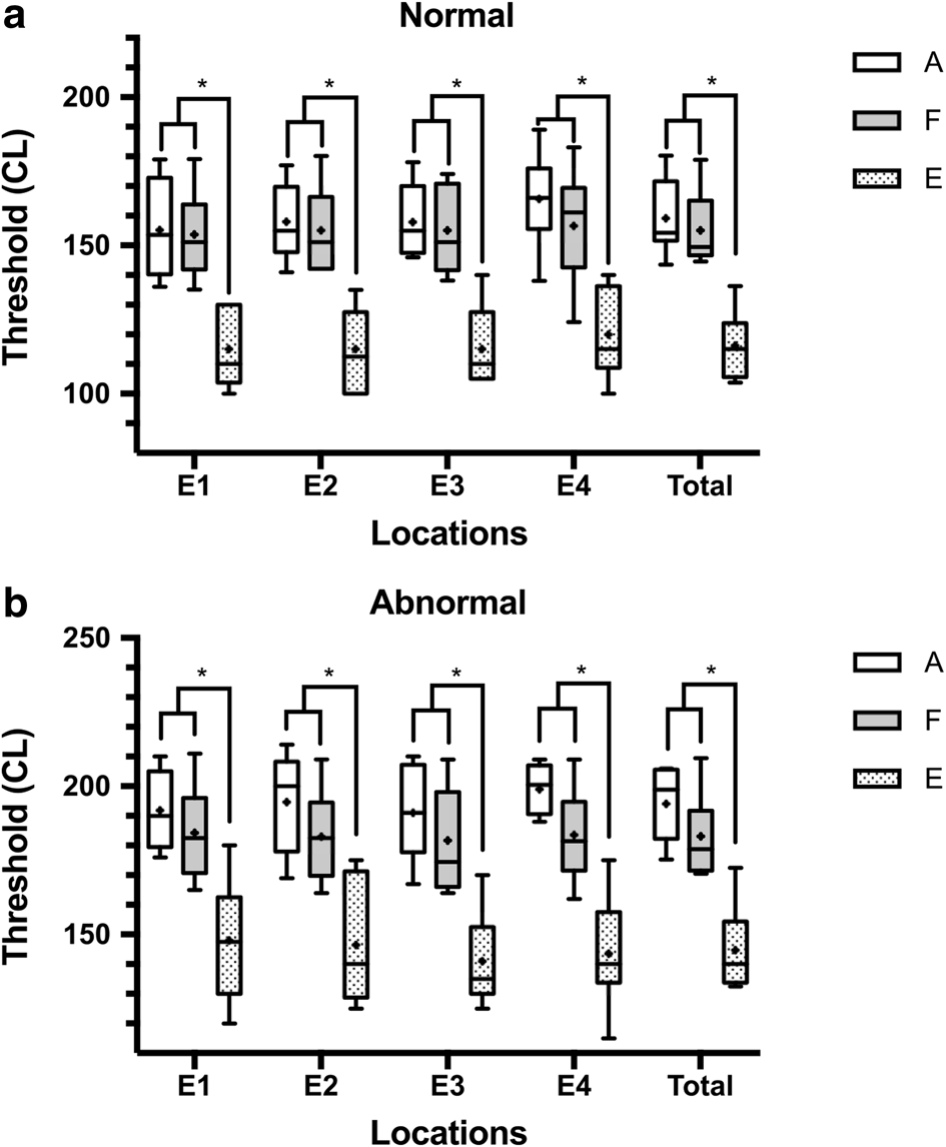
Comparison of the AP-ECAP and FM-ECAP thresholds under different FSANs. The AP-ECAP threshold was comparable to the FM-ECAP threshold under the “normal” FSAN, but both were significantly higher than the corresponding EABR threshold (**a**). The results under the “abnormal” FSAN were the same as those under the “normal” FSAN (**b**). *AP-ECAP* alternating polarity electrically evoked compound action potential, *FM-ECAP* forward-masking subtraction electrically evoked compound action potential, *EABR* electrically evoked auditory brainstem response, *FSANs* functional states of the auditory nerve, A: AP-ECAP, F: FM-ECAP, E: EABR, E1–E4: electrode 1–electrode 4, *p < 0.0001, Kruskal–Wallis by ranks version of one-way ANOVA, followed by Dunn’s method. The data are represented as the mean ± SEM

The average values of the AP-ECAP, FM-ECAP and EABR thresholds under the “normal” FSAN were 159.2, 155.0 and 116.3 CL, while those under the “abnormal” FSAN were 194.1, 183.1 and 144.7 CL, respectively (Table 1). There was no significant difference between the AP-ECAP and FM-ECAP thresholds at all electrodes under the “normal” and “abnormal” FSANs (*p* > 0.05) (Fig. 1a). However, there was a significant difference between the EABR thresholds and the two kinds of ECAP thresholds (*p* < 0.0001) (Fig. 1b). The mean gap between the AP-ECAPs and EABRs and between the FM-ECAPs and EABRs under the “normal” FSAN was 42.9 and 38.7 CL, respectively, while for those under the “abnormal” FSAN was 49.4 and 38.4 CL, respectively. These results suggested that the EABR thresholds were lower than the ECAP thresholds with the methods used in the present study.

**Table 1:** ECAP and EABR thresholds under different FSANs

	Electrode locations	AP-ECAP				FM-ECAP				EABR			
		Mean (CL)	SD (CL)	N	SE	Mean (CL)	SD (CL)	N	SE	Mean (CL)	SD (CL)	N	SE
Normal FSAN	E1	155.2	15.8	10	5.0	153.5	14.1	10	4.5	115.0	13.3	10	4.2
	E2	158.0	12.1	10	3.8	154.9	13.9	10	4.4	115.0	14.1	10	4.5
	E3	157.9	12.1	10	3.8	155.0	14.0	10	4.4	115.0	13.3	10	4.2
	E4	165.7	14.9	10	4.7	156.5	18.4	10	5.8	120.0	15.1	10	4.8
	Total	159.2	13.9	40	2.2	155.0	14.7	40	2.3	116.3	13.6	40	2.2
Abnormal FSAN	E1	191.8	13.1	10	4.2	184.3	15.0	10	4.8	148.0	19.5	10	6.2
	E2	194.6	16.0	10	5.1	183.0	15.2	10	4.8	146.5	20.1	10	6.4
	E3	191.0	15.7	10	5.0	181.7	16.6	10	5.3	141.0	14.9	10	4.7
	E4	199.0	7.7	10	2.4	183.5	15.5	10	4.9	143.5	17.5	10	5.5
	Total	194.1	13.4	40	2.1	183.1	15.0	40	2.4	144.7	17.6	40	2.8

**Fig. 2:**
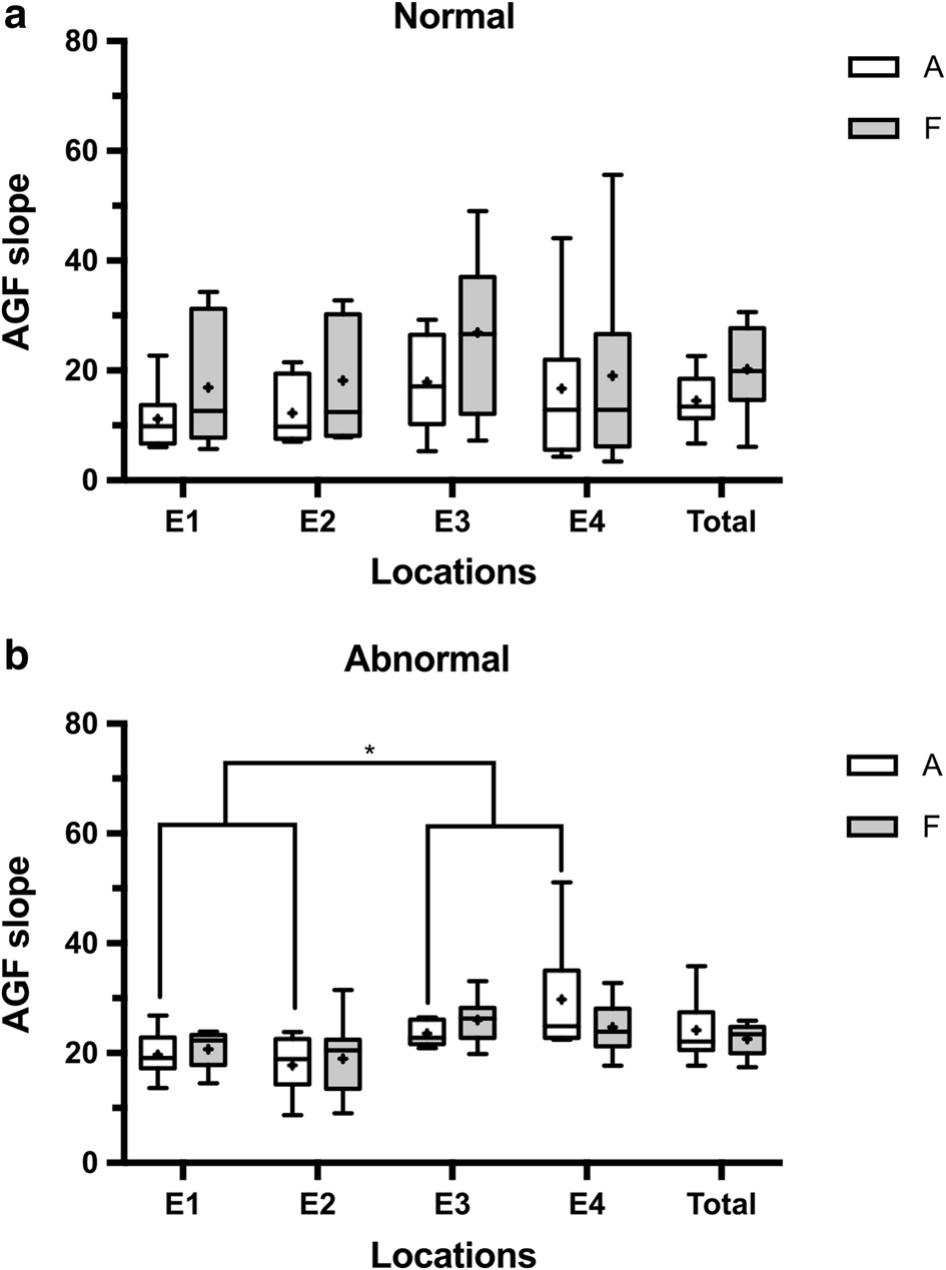
Comparison of the AGF slope values between the AP-ECAP and FM-ECAP under different FSANs. There was no significant difference in the slope values of the AGF between the AP-ECAP and FM-ECAP under the “normal” FSAN, and the slope values were similar across the electrodes within groups (**a**). However, there was a significant difference in the slopes of the AGF between electrodes 1 and 2 and electrodes 3 and 4 in the AP-ECAP under the “abnormal” FSAN (**b**). AGF: amplitude growth function, AP-ECAP: alternating polarity electrically evoked compound action potential, FM-ECAP: forward-masking subtraction electrically evoked compound action potential, FSANs: functional states of the auditory nerve, A: AP-ECAP, F: FM-ECAP, E1-E4: electrode 1–electrode 4, *p < 0.01, Kruskal–Wallis by ranks version of one-way ANOVA, followed by Dunn’s method. Data represent the mean ± SEM

The mean AGF slope values of the AP-ECAP and FM-ECAP were 14.5 and 20.3 under the “normal” FSAN, respectively, and 20.4 and 26.4 under the “abnormal” FSAN, respectively (Table 2). The slope values of the AGFs were not significantly different between the AP-ECAPs and FM-ECAPs under the “normal” FSAN or among the electrodes within groups (*p* > 0.05, Fig. 2a). However, there was an exception in the situation mentioned above under the “abnormal” FSAN. There was a significant difference between the AP-ECAP slope values of electrodes 1 and 2 and electrodes 3 and 4 (*p* < 0.01, Fig. 2b). These results indicated that the slope of the AP-ECAP may be more sensitive in reflecting the “abnormal” FSAN.

**Table 2:** AGF slopes

	Electrode locations	AP-ECAP				FM-ECAP			
		Mean	SD	N	SE	Mean	SD	N	SE
Normal FSAN	E1	11.2	5.6	10	1.8	16.9	11.1	10	3.5
	E2	12.2	5.7	10	1.8	18.2	10.7	10	3.4
	E3	17.9	8.8	10	2.8	26.9	14.6	10	4.6
	E4	16.7	14.3	10	4.5	19.1	19.3	10	6.1
	Total	14.5	9.4	40	1.5	20.3	14.3	40	2.3
Abnormal FSAN	E1	19.7	3.8	10	1.2	25.4	15.4	10	4.9
	E2	17.8	5.2	10	1.7	23.2	15.0	10	4.7
	E3	29.5	12.6	10	4.0	29.4	11.2	10	3.5
	E4	29.8	11.2	10	3.5	27.8	10.7	10	3.4
	Total	24.2	10.3	40	1.6	26.4	12.9	40	2.0

**Fig. 3:**
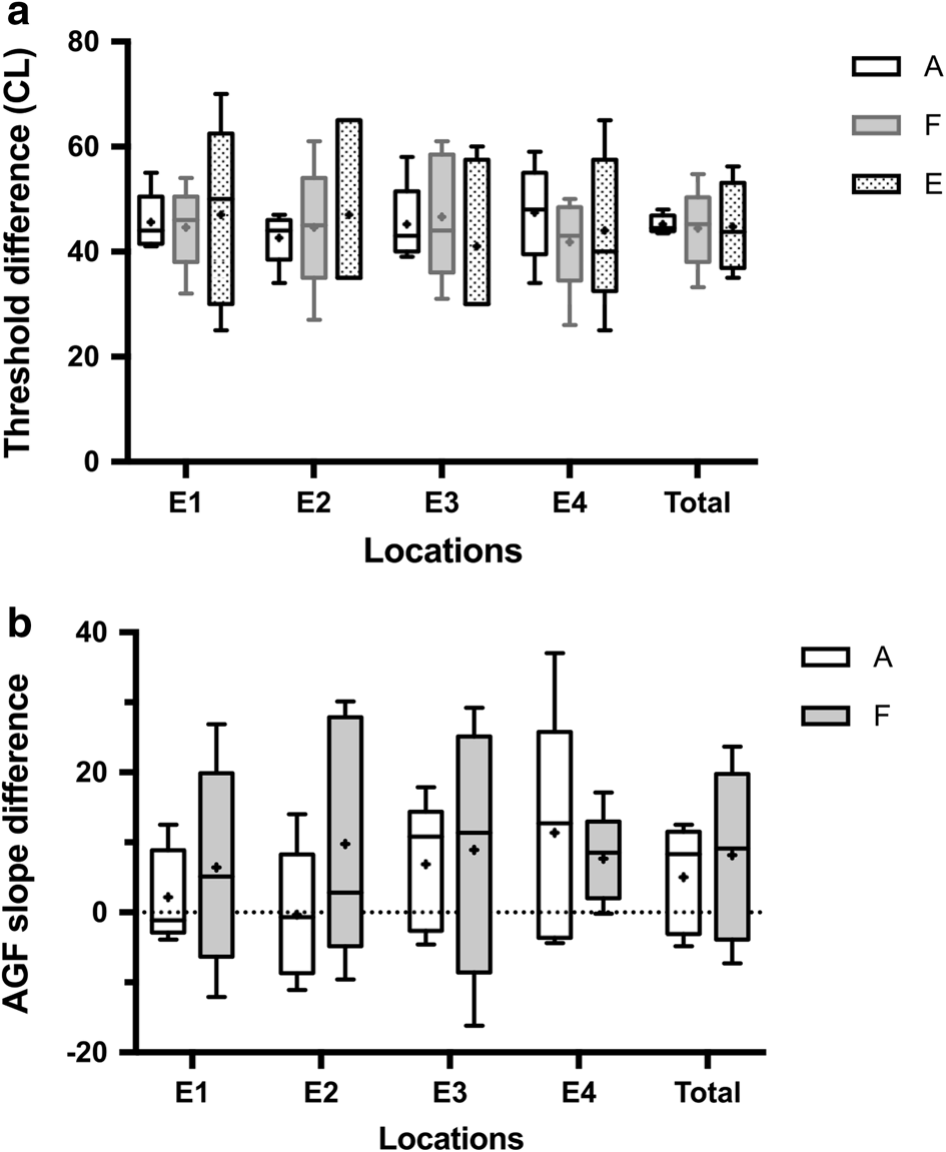
Comparison of the differences in the AP-ECAP, FM-ECAP and EABR thresholds as well as the corresponding slopes of the AGF under the “normal” and under “abnormal” FSANs for the AP-ECAP and FM-ECAP. There were no significant differences among the AP-ECAP, FM-ECAP and EABR thresholds under the “normal” and under “abnormal” FSANs (**a**), and similarly, there were not significant differences in the slope of the AGF for the AP-ECAP and FM-ECAP (**b**). *AP-ECAP* alternating polarity electrically evoked compound action potential, *FM-ECAP* forward-masking subtraction electrically evoked compound action potential, *EABR* electrically evoked auditory brainstem response, *AGF* amplitude growth function, *FSANs* functional states of the auditory nerve, A: AP-ECAP, F: FM-ECAP, E: EABR, E1–E4: electrode 1–electrode 4, Kruskal–Wallis by ranks version of one-way ANOVA, followed by Dunn’s method. The data are represented by the mean ± SEM

The gaps among the thresholds under the “normal” FSAN and under “abnormal” FSAN were not significantly different for the AP-ECAPs, FM-ECAPs and EABRs (*p* > 0.05), the mean values of which were 45.2, 44.4 and 44.8 CL, respectively (Table 3, Fig. 3a). Similarly, the gaps between the AGF slopes of the AP-ECAP and FM-ECAP were not significantly different (*p* > 0.05), as the mean values were 7.5 and 8.2, respectively (Table 4, Fig. 3b). These results suggested that the threshold gaps in the ECAP and EABR for the “normal” FSAN and “abnormal” FSAN were equally effective in reflecting the severity of the “abnormal” FSAN. The AGF slope gaps of the AP-ECAP and FM-ECAP were equally effective as well.

**Table 3:** Threshold gaps between normal and abnormal FSAN

Electrode locations	AP-ECAP				FM-ECAP				EABR			
	Mean (CL)	SD (CL)	N	SE	Mean (CL)	SD (CL)	N	SE	Mean (CL)	SD (CL)	N	SE
E1	45.6	5.6	5	2.5	44.6	8.0	5	3.6	47.0	17.5	5	7.8
E2	42.6	5.0	5	2.2	44.6	12.1	5	5.4	47.0	16.4	5	7.3
E3	45.2	7.5	5	3.4	46.6	12.0	5	5.4	41.0	15.2	5	6.8
E4	47.4	9.1	5	4.1	41.8	9.3	5	4.2	44.0	14.7	5	6.6
Total	45.2	6.7	20	1.5	44.4	9.8	20	2.2	44.8	14.9	20	3.3

**Table 4:** AGF slope gap between normal and abnormal FSAN

	Electrode locations	Mean	SD	N	SE
AP-ECAP	E1	3.3	6.3	5	2.8
	E2	2.0	7.5	5	3.3
	E3	11.1	17.1	5	7.6
	E4	13.6	21.2	5	9.5
	Total	7.5	14.2	20	3.2
FM-ECAP	E1	6.4	14.6	5	6.5
	E2	9.8	17.2	5	7.7
	E3	8.9	18.0	5	8.0
	E4	7.7	6.4	5	2.9
	Total	8.2	13.6	20	3.0

**Fig. 4:**
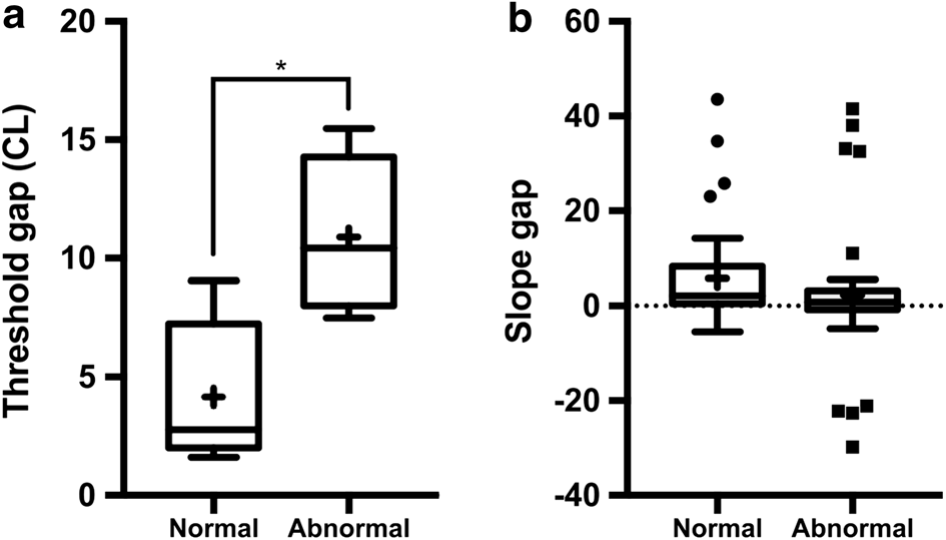
Comparison of the threshold gaps between the AP-ECAP and FM-ECAP as well as the corresponding slope gaps in the AGF under different FSANs. **a** The threshold gaps between the AP-ECAP and FM-ECAP were significantly smaller under the “normal” FSAN than under the “abnormal” FSAN. **b** AGF slope gaps of AP-ECAP and FM-ECAP under the “normal” FSAN were comparable to those under the “abnormal” FSAN. AP-ECAP: alternating polarity electrically evoked compound action potential, FM-ECAP: forward-masking subtraction electrically evoked compound action potential, AGF: amplitude growth function, FSANs: functional states of the auditory nerve, *p < 0.01, paired samples *t* test. The data are represented as the mean ± SEM

The threshold gaps between the AP-ECAP and FM-ECAP under the “abnormal” FSAN were significantly larger than those under the “normal” FSAN (*p* < 0.05), and the mean values were 11.0 and 4.2 CL for the “abnormal” and “normal” FSANs, respectively (Fig. 4a). The corresponding slope gaps of the AGF were comparable (*p* > 0.05), as the mean value was 5.8 under the “normal” FSAN and 2.2 under the “abnormal” FSAN (Fig. 4b). These results suggested that the “abnormal” FSAN augments the difference between the thresholds of the AP-ECAP and FM-ECAP. In other words, AP-ECAP is likely more sensitive in detecting changes in ECAP thresholds under the “abnormal” FSAN.

## Discussion

To explore the electrophysiological characteristics of ECAPs and EABRs under different FSANs, we compared and analysed the AP-ECAP, FM-ECAP and EABR thresholds as well as the slopes of the AGF for the AP-ECAPs and FM-ECAPs.

There was no significant difference between the AP-ECAP and FM-ECAP thresholds at any of the electrodes under the two different auditory nerve functions, but both thresholds were significantly higher than the EABR threshold by approximately 40 CL. Differences in the stimulation parameters, for example, the stimulation frequency, might account for this result. Next, the methods used to determine the threshold were also different: a gap larger than 50 µV between the P wave and N wave was considered a positive indicator for the ECAP threshold, while the minimum intensity of the electrical stimulation that elicited a V wave was considered the EABR threshold. Moreover, P waves, N waves and V waves occurred at different locations. In addition, the difference in the recording method also affected the results. ECAPs have near-field recording potentials in the vicinity of the auditory nerve with few superpositions, typically 50 superpositions. However, EABRs are far-field potentials recorded on the skin, requiring the superposition of 500–1000 signals [17].

When we compared the slope values of the AGF between electrodes 1 and 2 and electrodes 3 and 4 under the “abnormal” FSAN, we found a significant difference between them. Miller et al. found that the threshold of each nerve fibre to electrical stimulation was normally distributed and that the ECAP slope reflected the number of SGNs reaching the discharge threshold level with an increasing intensity of electrical stimulation [24]. These results indicated that AP-ECAPs and FM-ECAPs have an equal ability to reflect the number of SGNs at the level of the discharge threshold under the “normal” FSAN, but the slope values of the AGF for the AP-ECAP are more sensitive under the “abnormal” FSAN. Moreover, the distance between electrodes and the modiolus cochleae or the number of SGNs and auditory nerve fibres might be critical factors affecting these slopes [25].

There was no significant difference in the AP-ECAP, FM-ECAP and EABR thresholds or in the ECAP slope between the “normal” and “abnormal” FSANs”. This result suggested that the structure and function of the auditory nerve can be equally represented by the AP-ECAP, FM-ECP and EABR and that the abilities of these measures to reflect the number of SGNs at the level of the discharge threshold were not affected by the FSAN.

The difference between AP-ECAP and FM-CAP thresholds under the “normal” FSAN was significantly smaller than that under the “abnormal” FSAN. The difference between the AP-ECAP and FM-CAP slopes under the “normal” FSAN was comparable to that under the “abnormal” FSAN. The former result suggested that the AP-ECAP threshold was more sensitive than the FM-ECAP threshold under the “abnormal” FSAN and that it was more helpful to assess the damage to the FSAN. The difference in the algorithms used for processing ECAPs might account for this result: for the FM-ECAP approach, the refractory period in the auditory nerve was utilized to record the auditory nerve responses sequentially under a combination of four masking pulses and detection pulses. After four kinds of response components were subtracted, artefacts of electrical stimulation could be removed, and then the ECAP waveform elicited from detection pulses could be retained [26]. However, for the AP-ECAP approach, the auditory nerve was stimulated alternately by electrical impulses with opposite polarity. The polarity of the artefact was opposite, and the polarity of the nerve response did not change with the polarity of the stimulation signal. After superposition, most stimulation artefacts were cancelled out, and the ECAP components were enhanced [21]. Moreover, the latencies of the responses to anodic- and cathodic-leading pulses differ; averaging the responses together results in amplitudes that are smaller than that for either polarity alone due to temporal smearing [22, 27, 28]. This process led to a higher threshold for the AP-ECAP. The latter result suggested that the sensitivity of the AP-ECAP slope was equal to that of the FM-ECAP slope under the two kinds of FSANs. However, the results might not be accurate because of the small sample size and the large dispersion shown in the box plot.

## Conclusion

In this study, we established guinea pig models with cochlear implants and then analysed the electrophysiological characteristics of auditory nerves using the ECAPs and EABRs under different FSANs. Our results suggested that the AP-ECAP responded equivalently to the FM-ECAP in determining the threshold under the “normal” FSAN, but they were both significantly higher than those measured by the EABRs; the AP-ECAP was equal to the FM-ECAP in sensitivity under the “normal” FSAN, while the former was more sensitive than the latter in reflecting the FSAN under the “abnormal” FSAN or in different electrode locations.

## Methods

### Establishment of the guinea pig model with a unilateral cochlear implant

The following procedures, which involve Dunkin Hartley guinea pigs, were approved by the Ethics Review Board of the Eye and ENT Hospital at Fudan University. Guinea pigs aged 2–3 postnatal months and weighing 250–350 g were injected intraperitoneally with 0.2 ml of a 0.1% atropine solution, followed by 0.1 ml of a mixed tiletamine/zolazepam and xylazine hydrochloride solution per 100 g of their body weight (the 1.25 ml xylazine hydrochloride solution was added to the 5 ml tiletamine/zolazepam solution). Hairs on the post aurem were shaved after the guinea pigs were under deep anaesthesia, an incision was made, and the muscle and connective tissue on the auditory bulla were separated. The round window in the cochlea was then exposed, and the round window membrane was punctured with a sterile syringe needle. Electrodes (Listent Medical Tech. Co., Ltd.) were completely implanted into the cochlea through the round window unilaterally. The round window and auditory bulla were sealed with the muscles to fix the electrodes. The receiving stimulator and external electrode were implanted subcutaneously before the incision was sutured.

### Measurement of the FM-ECAPs, AP-ECAPs and EABRs

**Fig. 5:**
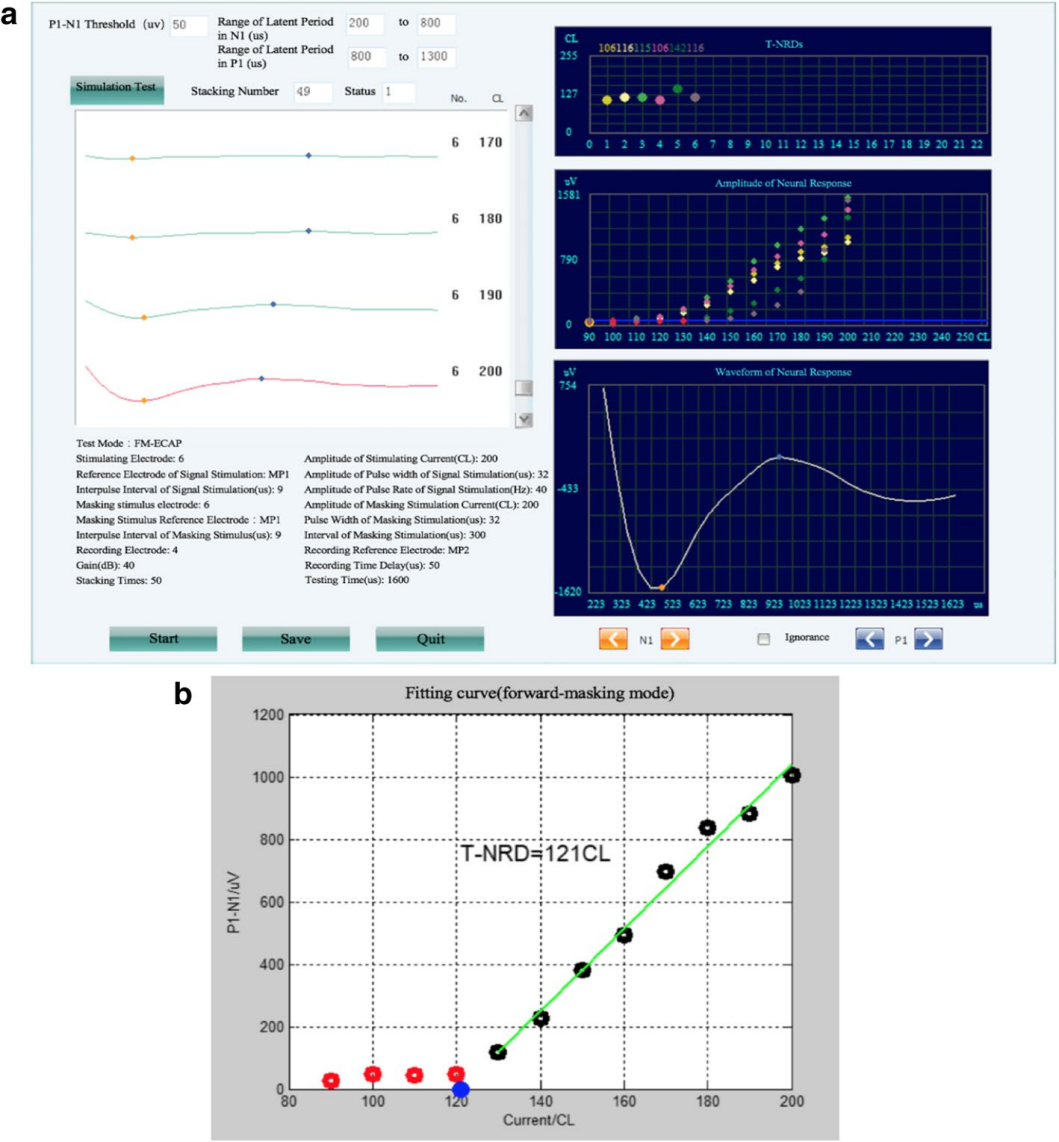
Interface diagram of the ECAP measurements and the AGF fitting curve. The left side of **a** shows the corresponding parameters of the ECAP measurements. The value in the top right corner of **a** is the corresponding ECAP threshold (CL) determined by electrodes 1–6 in turn. The middle section shows the amplitude of the auditory nerve responding to ES with an intensity from 90 CL to 200 CL, and the bottom section shows the difference between the peak and trough, which is the amplitude of the auditory nerve. **b** The AGF fitting curve obtained by running the original ECAP measurement file in MATLAB, from which we obtained the ECAP slope values. ECAP: electrically evoked compound action potential. *AGF* amplitude growth function

**Fig. 6:**
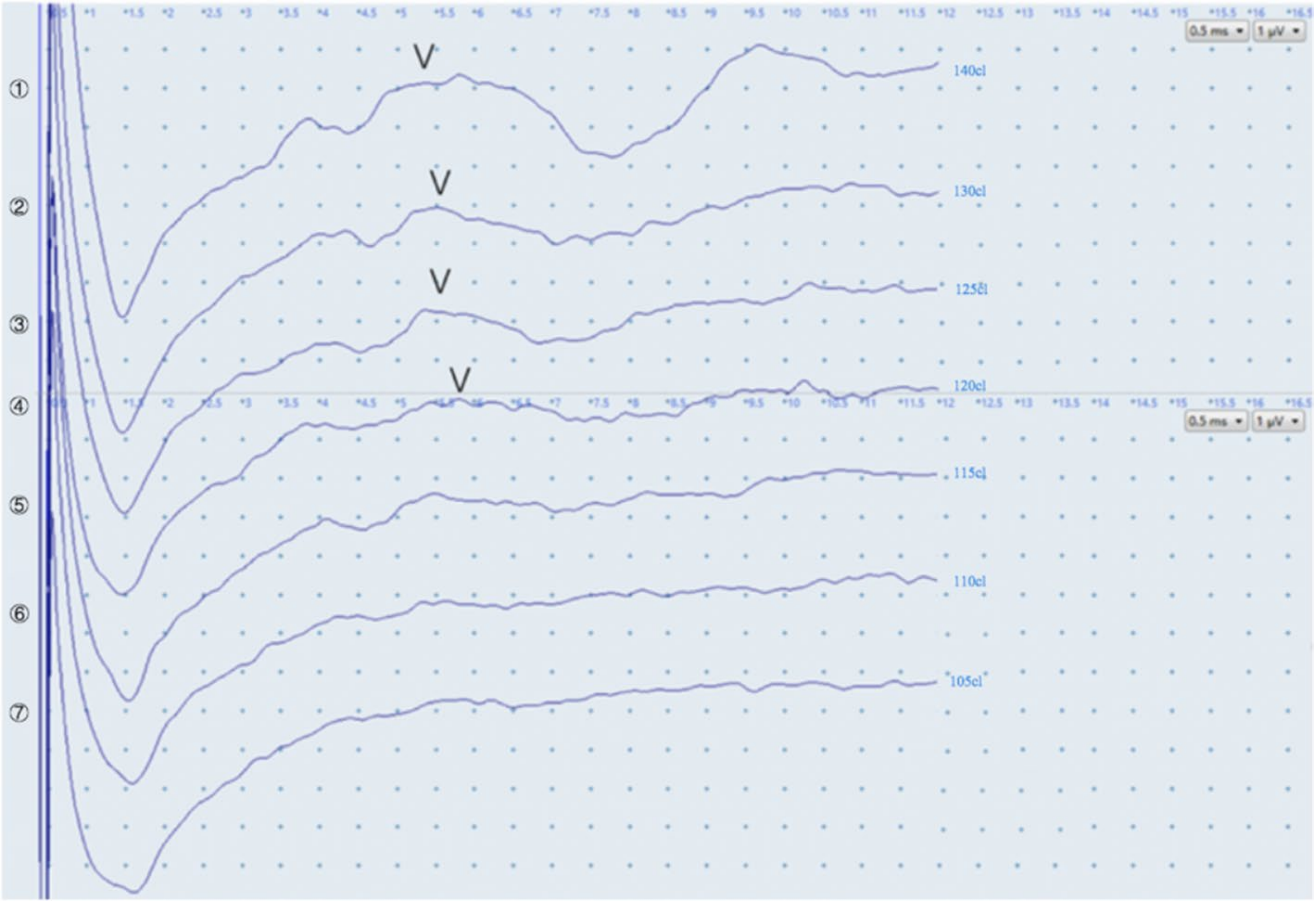
The waves of the EABR and the determination of the V wave. The V wave becomes obvious gradually with increasing ES intensity. We define the ES intensity as the EABR threshold when the first V wave occurs. The EABR threshold in the figure is 125 CL. EABR: electrically evoked auditory brainstem response

The electrodes were connected to an ECAP measurement instrument, which used MAP V3.00 software (Listent Medical Tech. Co., Ltd.). The impedances of the electrodes were measured first to ensure proper functioning. The FM-ECAP mode was selected, and the ECAP threshold of each electrode was measured successively (Fig. 5 ). Then, the AP-ECAP mode was selected, and the ECAP thresholds of electrodes 1–4 were measured successively. Subsequently, the EABR thresholds of electrodes 1–4 were measured sequentially using MAP V3.00 software and Neuro-MEP-Micro equipment (Neurosoft. Co., Ltd., RUS.). We obtained the EABR thresholds by determining V wave (Fig. 6).

### Application of ES to the guinea pig model with the electrodes

The electrodes were connected to the electrical pulse generator, which used V1.00 software (Listent Medical Tech. Co., Ltd.). The intensity of the ES (for electrodes 1–6) was set to be 6 dB higher than the FM-ECAP threshold. The electrical pulse generator was turned off after 4 h and then the AGFs of the AP-ECAPs and FM-ECAPs as well as the EABR waves were obtained again. We only recorded the data from electrodes 1–4 under the “abnormal” FSAN to reduce deviations in the measurements.

### Statistical analysis

All the original AP-ECAP and FM-ECAP data were processed in MATLAB to obtain the ECAP thresholds and slope values of the AGFs. The statistical analysis was performed by GraphPad Prism 7 (GraphPad Software, Inc.; CA, USA). The variables are reported as the mean ± SEM. The significance of the differences was determined by the paired samples *t* test or the Kruskal–Wallis by ranks version of one-way ANOVA, followed by Dunn’s method.

## Data Availability

The data sets used and/or analyzed during the current study are available from the corresponding author on reasonable request.

## Abbreviations

CI: Cochlear implant
SNHL: Severe-to-profound sensorineural hearing loss
ECAP: Electrically evoked compound action potential
EABR: Electrically evoked auditory brainstem response
FSAN: Functional states of auditory nerve
ES: Electrical stimulation
AGF: Amplitude growth function
AP-ECAP: Alternating polarity electrically evoked compound action potential
FM-ECAP: Forward-masking subtraction electrically evoked compound action potential
SGNs: Spiral ganglion neurons
